# Machine learning application for classification of Alzheimer's disease stages using ^18^F-flortaucipir positron emission tomography

**DOI:** 10.1186/s12938-023-01107-w

**Published:** 2023-04-29

**Authors:** Sang Won Park, Na Young Yeo, Jinsu Lee, Suk-Hee Lee, Junghyun Byun, Dong Young Park, Sujin Yum, Jung-Kyeom Kim, Gihwan Byeon, Yeshin Kim, Jae-Won Jang, for the Alzheimer’s Disease Neuroimaging Initiative

**Affiliations:** 1 Department of Neurology, Kangwon National University Hospital, 156, Baengnyeong-ro, Chuncheon, Gangwon 24289 Republic of Korea; 2 Department of Medical Informatics, Kangwon National University, Chuncheon, Korea; 3 Department of Data Science Research Center, Seoul National University Hospital, Seoul, Korea; 4 Department of Statistics, Kangwon National University, Chuncheon, Korea; 5 Department of Healthcare, Radiation Health Institute, Hydro & Nuclear Co., Ltd., Seongnam, Korea; 6 School of Medicine, Kangwon National University, Chuncheon, Korea; 7 Department of Medical Bigdata Convergence, Kangwon National University, Chuncheon, Korea; 8 Department of Psychiatry, Kangwon National University Hospital, Chuncheon, Korea

**Keywords:** Alzheimer's disease, Cognitive dysfunction, Positron-emission tomography, Tau proteins, Machine learning

## Abstract

**Background:**

The progression of Alzheimer’s dementia (AD) can be classified into three stages: cognitive unimpairment (CU), mild cognitive impairment (MCI), and AD. The purpose of this study was to implement a machine learning (ML) framework for AD stage classification using the standard uptake value ratio (SUVR) extracted from ^18^F-flortaucipir positron emission tomography (PET) images. We demonstrate the utility of tau SUVR for AD stage classification. We used clinical variables (age, sex, education, mini-mental state examination scores) and SUVR extracted from PET images scanned at baseline. Four types of ML frameworks, such as logistic regression, support vector machine (SVM), extreme gradient boosting, and multilayer perceptron (MLP), were used and explained by Shapley Additive Explanations (SHAP) to classify the AD stage.

**Results:**

Of a total of 199 participants, 74, 69, and 56 patients were in the CU, MCI, and AD groups, respectively; their mean age was 71.5 years, and 106 (53.3%) were men. In the classification between CU and AD, the effect of clinical and tau SUVR was high in all classification tasks and all models had a mean area under the receiver operating characteristic curve (AUC) > 0.96. In the classification between MCI and AD, the independent effect of tau SUVR in SVM had an AUC of 0.88 (*p* < 0.05), which was the highest compared to other models. In the classification between MCI and CU, the AUC of each classification model was higher with tau SUVR variables than with clinical variables independently, which yielded an AUC of 0.75(*p* < 0.05) in MLP, which was the highest. As an explanation by SHAP for the classification between MCI and CU, and AD and CU, the amygdala and entorhinal cortex greatly affected the classification results. In the classification between MCI and AD, the para-hippocampal and temporal cortex affected model performance. Especially entorhinal cortex and amygdala showed a higher effect on model performance than all clinical variables in the classification between MCI and CU.

**Conclusions:**

The independent effect of tau deposition indicates that it is an effective biomarker in classifying CU and MCI into clinical stages using MLP. It is also very effective in classifying AD stages using SVM with clinical information that can be easily obtained at clinical screening.

**Supplementary Information:**

The online version contains supplementary material available at 10.1186/s12938-023-01107-w.

## Background

Alzheimer’s dementia (AD) is the most common type of dementia with cognitive decline among older adults [1, 2]. In general, the progression of AD can be classified into three stages: cognitive un-impairment (CU), mild cognitive impairment (MCI), and AD. Currently, there is no standard treatment for AD; hence, the clinical treatment strategy is to reduce disease progression and establish biomarkers for early diagnosis and intervention.

Although pathological hallmarks of β amyloid (Aβ) deposition and tau neurofibrillary tangles (NFTs) are AD characteristics, it is known that tau burden is more strongly associated with cognitive dysfunction and neurodegeneration than Aβ accumulation [1, 3, 4]. Imaging biomarkers for AD have been defined by National Institute on Aging–Alzheimer's Association. They state that amyloid positron emission tomography (PET) can be used for Aβ and tau PET for NFTs [5]. PET images can visualize pathophysiological observations regarding molecular agglomeration and serve as potential outcomes of proof-of-concept clinical trials using experimental therapeutics [6–8]. Amyloid PET can provide pathological information for Aβ agglomeration. However, a flaw of the technique is that it even demonstrates Aβ accumulation 20 years before the diagnosis of AD, showing early saturation in the disease process [8–10]. On the contrary, tau PET scans based on the deposition of NFTs indicate a greater correlation with neurodegeneration and cognitive impairment, higher than amyloid PET [11, 12]. They can also directly reflect the characteristic pathology of AD [13]. Brain structural atrophy, observed by magnetic resonance imaging (MRI) as a conventional imaging biomarker, has a close relationship with tau deposition [14, 15]. The strength of tau PET images is that they can reveal tau accumulation patterns and specific deposits in focal regions of the brain, which can be demonstrated through Braak staging [16, 17]. Brain cross-sectional autopsies have revealed that AD associated with tau deposition begins in the medial temporal lobe (Braak stage I/II), migrates to the lateral temporal cortex and parts of the medial parietal lobe (stage III/IV), followed by migration to a large neocortical area (V/VI) [6, 18]. Therefore, if the morphological phenotype of tau is identified through in vivo molecular neuroimaging, such as tau PET, individuals with MCI and AD can be cognitively distinguished from those with CU.

According to the vast increase in medical imaging data, machine learning (ML) has been used for disease classification [19, 20]. This can include new tools transferred to the clinic for assistance in early diagnosis and prognosis. Although such classification has provided valuable information about AD biomarkers, a more substantial application of this technology can be for determining whether a CU patient will be classified with MCI. Thus, this study implements an ML framework for AD stage classification into CU, MCI, and AD using the Standard Uptake Value Ratio (SUVR) extracted from PET images. In addition, we demonstrate the utility of tau for AD stage classification.

## Results

### Participant characteristics

The characteristics of all participants in this study are presented in Table 1. The ML modeling included 199 participants, comprising 74, 69, and 56 patients in CU, MCI, and AD groups, respectively (Table 1). The overall mean age was 71.5 years, and that of CU, MCI, and AD groups were 69.3, 71.7, and 74.3 years, respectively. The study comprised 106 (53.3%) male and 93 (46.7%) female participants. There was a greater number of men in the MCI group (*n* = 45, 65.2%) than in the AD group (*n* = 35, 62.5%). The participants' average overall years of education was 16.5 years and the average mini-mental state examination (MMSE) score was 26.8. The AD group showed lower-than-average years of education and MMSE scores of 15.6 and 22.9, respectively, compared to the CU and MCI group, which had above-average years of education and MMSE scores. The average tau SUVR of all participants was 3.9 in the amygdala, 5.2 in the entorhinal cortex, 4.0 in the fusiform area, 13.3 in the temporal cortex, and 3.8 in the para-hippocampal cortex, indicating that there were differences in tau deposition according to the AD stage. Statistical analysis showed that all variables were useful for showing differences between the groups.

**Table 1:** Participant characteristics

	Total (*N* = 199)	CU (*N* = 74)	MCI (*N* = 69)	AD (*N* = 56)
Age (years)^*^	71.5 $\pm$ 7.4	69.3 $\pm$ 5.9	71.7 $\pm$ 7.8	74.3 $\pm$ 7.7
Sex^*^				
Female	93 (46.7%)	48 (64.9%)	24 (34.8%)	21 (37.5%)
Male	106 (53.3%)	26 (35.1%)	45 (65.2%)	35 (62.5%)
Education (years)^*^	16.5 $\pm$ 2.4	17.1 $\pm$ 2.3	16.6 $\pm$ 2.3	15.6 $\pm$ 2.5
MMSE^*^	26.8 $\pm$ 3.3	29.1 $\pm$ 1.0	27.5 $\pm$ 2.4	22.9 $\pm$ 2.8
Tau SUVR^*^				
Amygdala	3.9 $\pm$ 1.5	2.9 $\pm$ 0.6	4.1 $\pm$ 1.6	5.1 $\pm$ 1.4
Entorhinal	5.2 $\pm$ 1.9	4.0 $\pm$ 0.9	5.4 $\pm$ 1.8	6.6 $\pm$ 1.9
Fusiform	4.0 $\pm$ 1.6	3.3 $\pm$ 0.8	3.9 $\pm$ 1.6	5.0 $\pm$ 1.8
Temporal	13.3 $\pm$ 4.4	11.3 $\pm$ 2.1	13.0 $\pm$ 4.5	16.3 $\pm$ 4.9
Para-hippocampal	3.8 $\pm$ 1.1	3.2 $\pm$ 0.6	3.8 $\pm$ 1.2	4.6 $\pm$ 1.1
Amyloid status ( ±)	110/89	20/54	39/30	51/5

Values are presented as mean ± SD unless otherwise stated

^*^Indicates a variable that is significant between groups in the univariate analysis of variance and post-hoc analysis (*p* < 0.001). *CU* cognitively unimpaired, *MCI* mild cognitive impairment, *AD* Alzheimer’s disease, *SUVR* standard uptake value ratio

### Classification performance results of ML models

**Fig. 1:**
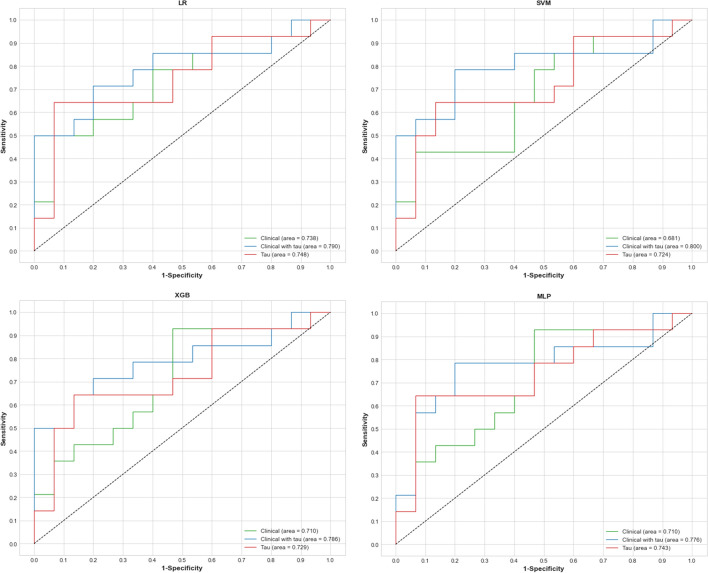
Receiver operating characteristic curve for classification between cognitively unimpairment and mild cognitive impairment

Tables 2–4 show the results of the performance of each model, including detailed metrics, such as accuracy, precision, recall, F1 score, and area under the receiver-operating characteristic (ROC) curve (AUC). Figure 1 shows the ROC curve for classification between CU and MCI of all models with each feature set (clinical variables, clinical and tau SUVR variables, and tau SUVR variables). Other results of ROC for classification between CU and AD and MCI and AD are presented in Additional file 1: Figs. S1, S2.

**Table 2:** Results of classification between CU and AD

	Accuracy	Precision	Recall	F1 Score	AUC
LR					
Clinical data	0.92 ± 0.00	0.85 ± 0.00	1.00 ± 0.00	0.92 ± 0.00	1.00 ± 0.00
Tau PET	0.88 ± 0.00	0.83 ± 0.00	0.91 ± 0.00	0.87 ± 0.00	0.95 ± 0.01
Clinical data with Tau	0.96 ± 0.00	0.92 ± 0.00	1.00 ± 0.00	0.96 ± 0.00	1.00 ± 0.00
SVM					
Clinical	0.96 ± 0.00	0.92 ± 0.00	1.00 ± 0.00	0.96 ± 0.00	1.00 ± 0.01
Tau	0.92 ± 0.00	0.91 ± 0.00	0.91 ± 0.00	0.91 ± 0.00	0.94 ± 0.01
Clinical with Tau	0.96 ± 0.00	0.92 ± 0.00	1.00 ± 0.00	0.96 ± 0.00	1.00 ± 0.00
XGB					
Clinical	0.94 ± 0.02	0.88 ± 0.04	1.00 ± 0.00	0.94 ± 0.02	0.99 ± 0.00
Tau	0.88 ± 0.04	0.86 ± 0.05	0.86 ± 0.05	0.86 ± 0.05	0.97 ± 0.01
Clinical with Tau	0.94 ± 0.02	0.88 ± 0.04	1.00 ± 0.00	0.94 ± 0.02	1.00 ± 0.01
MLP					
Clinical	0.91 ± 0.05	0.85 ± 0.08	0.98 ± 0.04	0.91 ± 0.06	0.99 ± 0.05
Tau	0.87 ± 0.03	0.83 ± 0.00	0.86 ± 0.05	0.84 ± 0.04	0.91 ± 0.03
Clinical with Tau	0.95 ± 0.01	0.90 ± 0.04	1.00 ± 0.00	0.95 ± 0.01	1.00 ± 0.02

*CU* cognitively un-impaired, *AD* Alzheimer’s disease, *LR* logistic regression, *SVM* support vector machine, *XGB* extreme gradient boosting, *MLP* multilayer perceptron, *AUC* area under the receiver operating characteristic curve. Values are presented as mean ± SD

In the classification of AD from CU (Table 2), the AUC of each classification model was the highest when clinical variables were used independently, with a similar effect when using clinical and tau SUVR variables. However, the independent use of tau SUVR variables was not more effective than others for classification. In the classification of MCI from CU (Table 3), the AUC of each classification model was higher with tau SUVR variables than with clinical variables used independently. In addition, the effect of using clinical and tau SUVR variables was the highest in each model for classification results. Multilayer perceptron (MLP) had the highest AUC of 0.75 for the independent use of tau SUVR variables, and the use of clinical and tau SUVR had the highest AUC in support vector machine (SVM), which was 0.81. In the classification of MCI from AD (Table 4), the effect of using tau SUVR variables was lower than that of others; AUC was 0.89 to 0.90. In extreme gradient boosting (XGB) and MLP models, the tau SUVR effect was higher than that in others. The effect of clinical and tau SUVR variables was highest in all models, with a mean AUC of 0.96. The XGB as a tree-based model, achieved a lower AUC value in all classification tasks than others. Overall, the classification results for clinical variables with tau SUVR showed the best performance in all metrics in all classification tasks.

**Table 3:** Results of classification between CU and MCI

	Accuracy	Precision	Recall	F1 Score	AUC
LR					
Clinical data	0.69 ± 0.00	0.65 ± 0.00	0.79 ± 0.00	0.71 ± 0.00	0.74 ± 0.00
Tau PET	0.76 ± 0.00	0.82 ± 0.00	0.64 ± 0.00	0.72 ± 0.00	0.75 ± 0.00
Clinical data with Tau	0.72 ± 0.00	0.68 ± 0.01	0.82 ± 0.04	0.74 ± 0.01	0.79 ± 0.00
SVM					
Clinical	0.76 ± 0.00	0.89 ± 0.00	0.57 ± 0.00	0.70 ± 0.00	0.68 ± 0.01
Tau	0.81 ± 0.02	0.91 ± 0.00	0.69 ± 0.00	0.78 ± 0.03	0.72 ± 0.02
Clinical with Tau	0.81 ± 0.02	0.82 ± 0.03	0.79 ± 0.04	0.80 ± 0.02	0.80 ± 0.02
XGB					
Clinical	0.69 ± 0.00	0.65 ± 0.00	0.79 ± 0.00	0.71 ± 0.00	0.71 ± 0.01
Tau	0.65 ± 0.06	0.69 ± 0.12	0.56 ± 0.22	0.61 ± 0.00	0.73 ± 0.00
Clinical with Tau	0.72 ± 0.04	0.69 ± 0.05	0.79 ± 0.08	0.73 ± 0.03	0.79 ± 0.02
MLP					
Clinical	0.63 ± 0.06	0.66 ± 0.10	0.55 ± 0.06	0.56 ± 0.17	0.71 ± 0.06
Tau	0.75 ± 0.02	0.83 ± 0.06	0.61 ± 0.04	0.70 ± 0.01	0.74 ± 0.01
Clinical with Tau	0.77 ± 0.05	0.75 ± 0.07	0.80 ± 0.04	0.77 ± 0.05	0.78 ± 0.05

*CU* cognitively un-impaired, *MCI* mild cognitive impairment logistic regression, *SVM* support vector machine, *XGB* extreme gradient boosting, *MLP* multilayer perceptron, *AUC* area under the receiver operating characteristic curve. Values are presented as mean ± SD

**Table 4:** Results of classification between MCI and AD

	Accuracy	Precision	Recall	F1 Score	AUC
LR					
Clinical data	0.88 ± 0.00	0.84 ± 0.00	0.90 ± 0.00	0.88 ± 0.00	0.94 ± 0.00
Tau PET	0.84 ± 0.00	0.82 ± 0.00	0.82 ± 0.00	0.82 ± 0.00	0.90 ± 0.01
Clinical data with Tau	0.89 ± 0.02	0.88 ± 0.04	0.88 ± 0.05	0.86 ± 0.02	0.95 ± 0.01
SVM					
Clinical	0.96 ± 0.00	0.92 ± 0.00	1.00 ± 0.00	0.96 ± 0.00	0.94 ± 0.00
Tau	0.88 ± 0.00	0.90 ± 0.00	0.83 ± 0.00	0.85 ± 0.00	0.89 ± 0.00
Clinical with Tau	0.96 ± 0.00	0.93 ± 0.04	0.98 ± 0.04	0.96 ± 0.00	0.95 ± 0.01
XGB					
Clinical	0.88 ± 0.00	0.90 ± 0.00	0.83 ± 0.00	0.85 ± 0.00	0.95 ± 0.01
Tau	0.79 ± 0.02	0.86 ± 0.01	0.66 ± 0.04	0.71 ± 0.03	0.90 ± 0.00
Clinical with Tau	0.89 ± 0.04	0.89 ± 0.01	0.85 ± 0.10	0.84 ± 0.06	0.97 ± 0.01
MLP					
Clinical	0.83 ± 0.10	0.84 ± 0.10	0.78 ± 0.16	0.78 ± 0.13	0.95 ± 0.11
Tau	0.82 ± 0.05	0.92 ± 0.07	0.70 ± 0.09	0.75 ± 0.07	0.90 ± 0.05
Clinical with Tau	0.86 ± 0.05	0.90 ± 0.05	0.82 ± 0.11	0.83 ± 0.07	0.97 ± 0.06

*MCI* mild cognitive impairment, *AD* Alzheimer’s disease, *LR* logistic regression, *SVM* support vector machine, *XGB* extreme gradient boosting, *MLP* multilayer perceptron, *AUC* area under the receiver operating characteristic curve .Values are presented as mean ± SD

### An explanation for feature importance

**Fig. 2:**
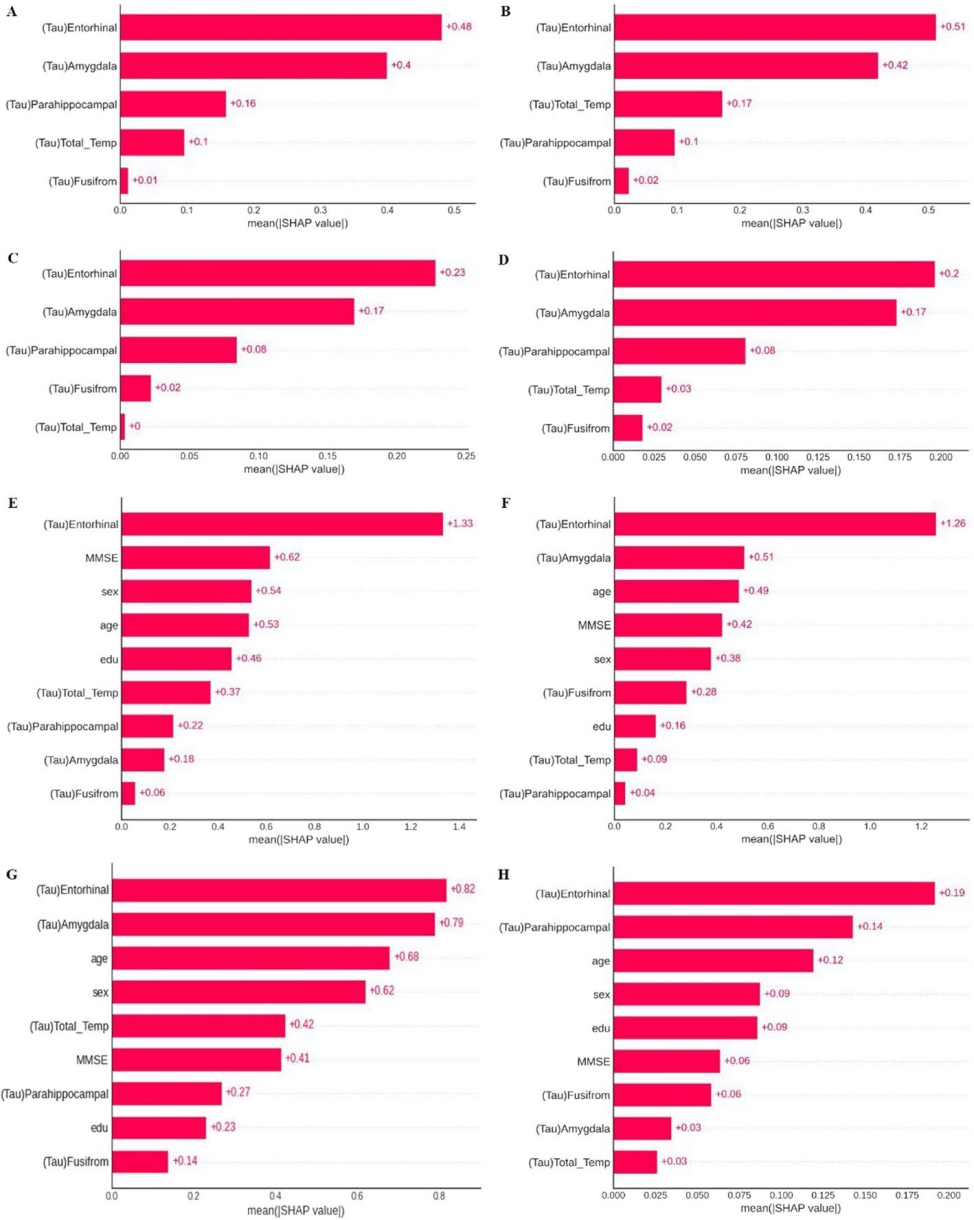
Feature importance results based on the explainable Shapley Additive Explanations method. **a**–**d** Are the results for the importance of the tau standard uptake value ratio (SUVR) features, considering the trade-offs between the features. **e**–**h** Are the results for clinical variables with tau SUVR features

Figure 2 shows the feature importance calculated by the Shapley value of each ML model for classification between CU and MCI, which can explain the features with high characteristics. Other classification tasks are presented in Additional file 1: Figs. S3, S4. We excluded the results for feature importance using clinical variables for classification, as the MMSE score had a high effect on the results in all models for all tasks. In the classification between MCI and CU and that between AD and CU using tau SUVR alone, the amygdala and entorhinal cortex SUVR greatly affected the performance. In the classification of MCI from AD, the para-hippocampal and temporal cortex SUVR greatly affected the performance.

The highest effect of MMSE score on performance results for classification between MCI and CU and between MCI and AD was observed when using clinical variables. After considering the MMSE score, the amygdala, and entorhinal and temporal lobe SUVR showed high classification accuracy for distinguishing AD from CU. Similarly, the fusiform, entorhinal, and para-hippocampal cortex SUVR effectively classified MCI from CU. In classification of MCI from CU, tau SUVR of the entorhinal cortex and amygdala had a higher effect on model performance than clinical variables, including MMSE score.

## Discussion

In this study, we investigated the effects of baseline tau SUVR extracted from ^18^F-flortaucipir PET scans and basic clinical variables of AD on classification prediction. The investigation was carried out using multiple representative ML, such as logistic regression (LR), SVM and XGB, and basic neural network, such as MLP algorithms. The used clinical variables, such as age, sex, education, and MMSE score, which are indicators easily obtainable from the screening stage of AD clinical trials or from hospital visits of outpatients. The variables were used alone or in combination to compare the performance between ML models. We identified some features which have major effects on results through importance. In addition, we demonstrated that combining clinical variables with tau SUVR improved model performance. We confirmed that the effect of independent use of tau SUVR has a remarkable performance for classification between CU and MCI.

The combination of clinical variables and tau SUVR showed the highest performance in all metrics (accuracy, precision, recall, F1 score, AUC) when comparing ML models for AD stage classification tasks. The classification task for each model showed higher accuracy with clinical variables with tau SUVR than with clinical data or tau SUVR alone. In addition, the uniqueness of the independent tau SUVR was best demonstrated for classification between CU and MCI (Table 3). The usefulness of independent utilization of tau SUVR was that it showed a high performance for all assessment metrics compared to clinical variables in the classification between MCI and CU, which are two stages that are not easy to classify clinically. These results demonstrate the superior effect of tau SUVR in the classification between CU and MCI stages and its potential utilization as a biomarker for early stage classification, enabling the identification of individual stages. However, the two classification tasks between MCI and AD or AD and CU showed a slightly more effective performance of clinical variables than tau SUVR alone. It indicates high dependence on influential neuropsychological information, such as MMSE score, showing a significant difference between the groups. It means that it cannot be easily classified with basic clinical information for MCI and CU, meaning that tau deposition levels are essential and effective for early stage AD classification.

Another interesting point in this study is that each ML algorithm was explained by a sophisticated and complicated feature importance using the shapley additive explanations (SHAP) method than the conventional method (Fig. 2, Additional file 1: Figs. S3, S4). This study used the SHAP method to calculate the importance of tau SUVR characteristics in affecting the ML classifiers' performance results. The effect of offsetting and those between all variables, including clinical variables, were investigated, as well as the presentation of results. In general, the artificial intelligence (AI) model is known as a black box, as the output prediction or decision-making of the model cannot be interpreted or explained. Recently, many studies have been conducted to interpret AI models to explain the behavior of AI. Especially in tabular data, the model explanation can be represented as feature importance, which denotes the individual influence of each feature on the output prediction. Compared to general feature importance, SHAP value-based feature importance has a detailed explanation by consideration of offsets between all variables for model result calculation based on permutation calculation [21, 22]. A surrogate model is first trained to approximate the original model to compute the SHAP values. The surrogate model is a simpler, interpretable model that is easier to explain. SHAP values are then computed for each feature by evaluating the difference between the predictions of the surrogate model and the predictions when that feature is set to its background value (typically the mean or median of the training data). The conventional feature importance is likely to suggest different importance depending on the parameter influence as the model iteration is performed. Therefore, in this study, the SHAP method was used to calculate the importance of tau SUVR characteristics affecting performance results of the ML classifier, and the effect of offsetting and those between all variables, including clinical variables, were investigated with the presentation of results.

This study has some limitations. First, conventional algorithms, such as logistic classification and SVM, which are the most commonly used, had higher performance results in five metrics than complex algorithms, such as XGB and MLP. The highest accuracy in MCI from CU for tau SUVR alone was 81%, achieved by SVM, and the accuracy of XGB and MLP for the same features was 65% and 75%, respectively. The relatively low accuracy in this study may be due to the small size of the data set used in this study. The Alzheimer’s Disease Neuroimaging Initiative (ADNI) is a very useful public database containing data from approximately 1,700 participants and has been used as a data set in over 3500 publications since 2004 [23]. We found a substantial amount of patient data, with 363 CU and 194 MCI participants, but only 59 were patients with AD. Thus, we randomly selected participants in CU and MCI to match the data count balance for ML algorithms and statistical analysis. Therefore, the total number of participants is very small. Second, the lack of participants caused another limitation. For this study, we attempted a comparison of the results with those of amyloid PET SUVR. However, as a result of extracting patients who provided amyloid PET information at baseline, the total number of participants was reduced to such an extent that analysis was difficult. It is known that Aβ is deposited throughout the brain as neurodegenerative and cognitive decline progresses and is expressed through PET scans. In contrast, tau level is sensitive to early neurodegeneration and cognitive decline and is expressed by PET scan through isthmus deposition [24, 25]. As amyloid deposition precedes tau accumulation, we only provided the status of amyloid at baseline at the time of tau acquisition. In addition, amyloid deposition precedes tau accumulation, and the accumulation target regions of the two proteins are also designated differently [26]. If more patients are secured in the future, it will be possible to prove the usefulness of tau compared with amyloid based on the model conducted in this study under the same environment and conditions. In addition, direct comparison with previously conducted amyloid studies is expected to be possible [27, 28]. Third, the SHAP interpretation we used in this study has a potential issue with the SHAP method: the attribution of feature importance is normally based on random permutations, whereas for a particular predictor, the samples are permuted randomly. However, this approach can have disadvantages, such as a high correlation among predictors. When the features are highly correlated, the SHAP values may not accurately reflect the true importance of each feature. In further work, we will collect more participants and apply the Kernel SHAP method, which uses a weighted sampling approach to compute the SHAP values that account for the correlation among features.

## Conclusions

In this study, we demonstrated that the tau deposition level is very effective in classifying AD stages when used together with clinical information that can be easily obtained at clinical screening. In addition, the independent effect of tau deposition level indicates that it effectively classifies CU and MCI into clinical stages.

## Methods

### Participants

All participants were enrolled in the ADNI (adni.loni.usc.edu) on March 03, 2022. Tau-PET scans were performed at a baseline of 199 enrolled participants (74 in the CU group, 69 in the MCI group, and 56 in the AD group). Information such as age, sex, education, MMSE score, tau-PET SUVR, and diagnostic results were acquired. All participants in the CU group had clinical dementia rating (CDR) scores of 0, which allowed them to be distinguished from those with MCI and AD. The patients with MCI did not meet the dementia criteria and were evaluated based on an objective memory impairment determination. All participants with MCI had MMSE scores ≥ 24 and CDR scores ≥ 0.5. In addition, a score that indicated impairment on the delayed recall of Story A from the Wechsler Memory Scale-Revised (≥ 16 years of education: < 11; 8–15 years of education: ≤ 9; 0–7 years of education: ≤ 6) assessment was applied. All patients who met the criteria for AD had CDR scores of ≥ 1, and Wechsler Memory Scale-Revised scores as follows: ≥ 16 years of education: ≤ 8; 8–15 years of education: ≤ 4; 0–7 years of education: ≤ 2 (Table 5). This study was performed according to the guidelines and regulations of the institutional review board and approved.

**Table 5:** Classification of ADNI participants into CU, MCI and AD groups

	CU	MCI	AD
Subjective memory complaint	None	Yes	Yes
MMSE score	≥ 24	≥ 24	Between 20 and 24 (Exceptions for 24 and 25 for participants with less than 8 years of education)
CDR	CDR = 0 Memory Box score must be 0	CDR = 0.5 Memory Box score of at least 0.5	CDR = 0.5 or 1.0
Logical memory score	≥ 9 for 16 or more years of education ≥ 5 for 8–15 years of education ≥ 3 for 0–7 years of education	≤ 8 for 16 or more years of education ≤ 4 for 8–15 years of education ≤ 2 for 0–7 years of education	≤ 8 for 16 or more years of education ≤ 4 for 8–15 years of education ≤ 2 for 0–7 years of education
General cognition and functional status	Cognitively normal based on the absence of significant impairment in cognitive functions or activities of daily living	General cognition and functional performance sufficiently preserved, such that a diagnosis of dementia cannot be made	NINCDS/ADRDA criteria for probable AD

*ADNI* Alzheimer’s Disease Neuroimaging Initiative ; *CU* cognitively unimpaired ; *MCI* mild cognitive impairment ; *MMSE* Mini-Mental State Examination ; *CDR* The Clinical Dementia Rating Scale

*NINCDS/ADRDA* National Institute of Neurological and Communication Disorders and Stroke/Alzheimer’s Disease and Related Disorders Association. This table was adapted and modified from the procedure manuals for ADNI3 available at http://adni.loni.usc.edu/methods/documents/

### Data acquisition and pre-processing

Min–max normalization was conducted for all variables to improve the ML model performance. PET images were used to extract SUVR. The ^18^F-flortaucipir 3D dynamic PET scan images for all individual patients acquired using “Coreg, Avg, Std Img and Vox Siz, and Uniform Resolution” in ADNI. All PET images were acquired through a 30-min scan, from 75 to 105 min after intravenous injection of ^18^F-flortaucipir radioisotope (RI), 370 mBq (10.0 mCi) ± 10% radioactivity, considering the weight of each patient. Partial volume correction was conducted as a post-process. The anterior–posterior axis in the brain was rearranged to be parallel to the anterior commissure-posterior commissure line, and spatial standardization was based on the Montreal Neurologic Institute atlas. Considering some information from the images might have been lost during preprocessing, interpolation was performed to minimize the existing information. Intensity normalization was performed for radioisotope uptake for each brain region based on cerebellar gray matter. Further details for the processing of tau-PET data can be found in other related studies [14, 29–32].

### SUVR measurement and definition

The measured SUVR and region of interest (ROI) definition using PET images for all participants were obtained after acquiring mask images through co-registration with T1 MRI of individual patients. Co-registration was conducted using Statistical Parametric Mapping (SPM 8, Wellcome Department of Imaging Neuroscience, London, United Kingdom), pairing each PET scan timepoint with individual MRI images. The ROI composed of 68 cortical and 12 subcortical regions were segmented through Freesurfer (version. 7.1.1) based on the Desikan–Killiany atlas to calculate SUVR. The cerebellar cortex was used as the reference region, and the quantified SUVR value was corrected for each region defined by the Braak stage. The volume of corresponding brain tissue was used as the weight [3, 4, 13]. The feature used in the ML model was selected by matching the region, including the I/II region of the entorhinal cortex, with the Braak stage based on established theory-driven ROIs that explain the pathological progression of tau with the progression of AD up to stage I/IV [11, 31, 33].

Mean radioactivity was acquired from each ROI using corrected images. The cerebellar gray matter (GM) was set as a reference region to specify the ROI. The SUVR, evaluated as the ratio of the activity of target ROI to that of reference ROI (cerebellar GM) and six cortical regions (frontal, temporal and temporal, occipital, parietal, and anterior and posterior cingulate cortex), was calculated using the unweighted mean [16, 34, 35].

### Amyloid status

The amyloid status of all participants was determined with a threshold using either FBB or 18F-AV45, which are detailed elsewhere [36, 37]. Moreover, we classified each participant as Aβ-positive PET scan on observing a global standardized uptake value ratio (SUVR) > 1.11 for the ^18^F-florbetapir. For ^18^F-florbetaben, tracer uptake was assessed according to the regional cortical tracer uptake system in four brain regions (frontal cortex, posterior cingulate cortex/precuneus, parietal cortex, and lateral temporal cortex) and the cutoff value was 1.1.

### Machine learning methods

#### Logistic regression

As a form of supervised learning in ML, LR is a conventional probabilistic statistical model for classification that has been broadly used across disciplines in medical sciences [38]. The LR models the relation between a continuous independent and a categorical dependent variable. It can predict and classify a sample to a group as a probability value between 0 and 1 which learns the relationship between the independent variables × 1, × 2, ···, *xn*, and the dependent variable *y* as a specific function. In other words: *y* = (*w*1 × 1 + ⋯ + *wnxn*), where *w*1, ···, *wn* are trainable parameters and σ is the sigmoid function, such that σ(*t*) = 1/(1 + *e* − *t*). In linear regression, the predicted dependent variable falls within the range [-∞, ∞]. The LR to classify binary tasks becomes possible by application of the sigmoid function, which always returns a probability in the range of [0, 1].

#### Support vector machine

The linear discriminant function of SVM is an algorithm that allows classification by defining a decision hyperplane in two or multiple dimensions [39]. SVM, a commonly used algorithm in ML for classification tasks, uses the optimal hyperplane, which maximizes the gap between two groups. The points where each datum is distributed and the distance between the classes of parallel hyperplanes passing the support vector through the optimal separating hyperplane are used for classification using margins, such as hard- or soft-margin, which can be determined by maximizing. The hyperplane is not unique and can be estimated by maximizing the classifier's performance, i.e., the classifier's ability to operate satisfactorily with any data.

#### XGB

The XGB is a tree-based algorithm that uses a boosting technique to lower the error value by bundling several classification and regression trees [40]. As an ensemble method, it can continuously train weak learners and strengthen them by combining weak classifiers. The data which weak learners fail to classify are given more weight when the next strong learner trains. Thus, the classifier can improve performance while focusing on previously misclassified data. The final results of this ensemble model are made by combinations of predictions from all weak and strong learners. The decision trees are expanded horizontally (i.e., levelwise) to reduce their depth. Some methods are applied to prevent overfitting and parallel algorithms, thereby gaining higher accuracy and lesser time cost. In addition, to prevent overfitting, a regularized learning objective can add to the loss function by restricting the increase in the model complexity.

#### MLP

The MLP consists of multiple perceptron layers and is a feed-forward artificial neural network used in diverse fields. MLP, composed of two or three to thousands of layers, can explain more information [41]. It can also be used to implement available classifier algorithms for distinguishing data that are not linearly separable. However, as MLP can also lead to overfitting because of several layers, activation functions, such as sigmoid, hyperbolic tangent, rectified linear unit, or softmax function, etc., can be applied in the model according to the condition [42–44]. In addition, optimizers in MLP, such as stochastic gradient descent, momentum, root mean square propagation, or Adam, decide the method by which the model learns from the loss calculated from input data. The learning rate affects the learning procedure, such as converging speed and direction of learning [45, 46].

#### Data split and validation

Of the total data, 80% was used for training, and the remaining data were used as test data. Validation data was used as 20% of the total training set. Of the selected ADNI participant’s data (i.e., entire participants), 20% was removed entirely from the cross-validation-based estimation of hyperparameter values for each of the four classification methods. In addition, stratified k-fold cross-validation (*k* = 3) was performed to avoid label distortion that might occur during model generation and to maintain model stability. The stratified k-fold cross-validation technique is similar to the regular k-fold cross-validation, except that stratified sampling is used instead of random sampling. In addition, for all models and classification tasks, the results were presented by performing five repeated iterations under the same conditions as above.

#### Informative feature explanation based on shapley additive explanations

We explained the classification prediction of our model with SHAP [47, 48], which is one of the methods to explain the model with feature importance and is inspired by the concept of coalition game theory by replacing “player” in coalition game theory with “data feature” in tabular data. In the ML context, the agents correspond to the features of the data, and the goal is to explain the model's prediction. The explanation is deduced as a linear function of the feature. The original model f is explained with a surrogate model g. The surrogate model g is defined as

$$\mathrm{g}\left(\mathrm{z}\right)= {\phi }_{0}+ \sum_{j=1}^{M}{\phi }_{j}{z}_{j}$$

where *z*' stands for the coalition vector, the attribution of feature *j*, and *M* is the maximum number of features.

#### Evaluation performance

Four metrics, such as accuracy, recall, precision, and F1 score, were used to evaluate the model performance. As this study focused on the accurate classification of CU vs. AD and CU vs. MCI, the true positive (TP) metric was mainly established for the overall performance evaluation of the classification model. In Eqs. 1–4, TP refers to the number of patients correctly predicted as CU, and false positive (FP) is the outcome predicted as AD or MCI.

1 $$\mathrm{Accuracy}= \frac{TP+TN}{TP+TN+FP+FN}$$

2 $$\mathrm{Precision}= \frac{TP}{TP+FP}$$

3 $$\mathrm{Recall}= \frac{TP}{TP+FN}$$

4 $$F1\mathrm{ Score}= 2\times \frac{\mathrm{Precision}\times \mathrm{Recall}}{\mathrm{Precision}+\mathrm{Recall}}$$

### Statistical analysis

Chi-squared ($\chi$^2^) test and analysis of variance (ANOVA) were used to confirm the difference in ratio and mean among the three groups (CU, MCI, AD) by variable. After conducting Levene's test for checking the equality of variances, ANOVA was performed to test the difference in the means among the three groups. In addition, if the assumption of equal variance was not satisfied, Welch's ANOVA was performed to test the mean difference among groups.

### Tools

**Fig. 3:**
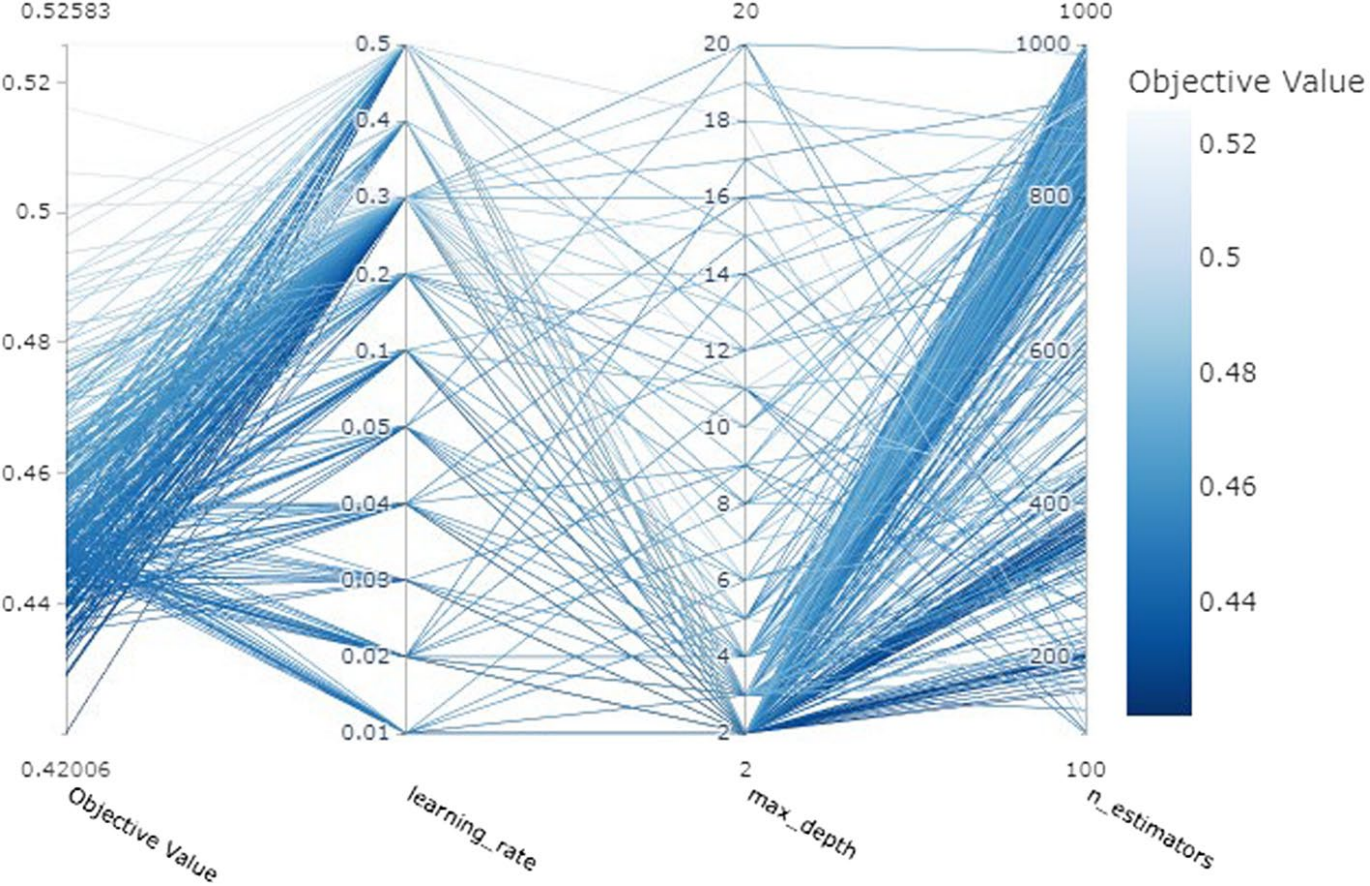
Hyper-parameter optimization for machine learning model framework using Optuna. The Optuna, as a method library for hyper-parameter tuning, can be used to derive the most optimized model performance. The higher density of presented lines refers to a combination of parameters that can create high performance

All processing for this study was done using Python (3.7.0, Python Software Foundation, Beaverton, OR, USA) and SAS (9.4, SAS Institute, Cary, NC, USA) software. ML modeling was performed using sci-kit-learn version 0.24.2. The hyper-parameter tuning was performed using Optuna, a hyper-parameter optimization framework (Fig. 3)[49].

## Supplementary Information

**Additional file 1.** Fig. 1: Receiver operating characteristic curve for classification between cognitively unimpairment and Alzheimer’s dementia. Fig. 2: Receiver operating characteristic curve for classification between mild cognitive impairment and Alzheimer’s dementia. Fig. 3. In between cognitive unimpairment and Alzheimer's disease, feature importance results based on the explainable Shapley Additive Explanations method. A–D Are the results for the importance of the tau standard uptake value ratio (SUVR) features, considering the trade-offs between the features. e–h are the results for clinical variables with tau SUVR features. Fig. 4. In between mild cognitive impairment and Alzheimer's disease, feature importance results based on the explainable Shapley Additive Explanations method. A–D Are the results for the importance of the tau standard uptake value ratio (SUVR) features, considering the trade-offs between the features. e–h are the results for clinical variables with tau SUVR features.

## Data Availability

All ADNI data used in this study is available through the ADNI website (http://adni.loni.usc.edu/data-samples/access-data/).

## Abbreviations

AD: Alzheimer’s dementia
CU: Cognitive un-impairment
MCI: Mild cognitive impairment
SUVR: Standard uptake value ratio
PET: Positron emission tomography
LR: Logistic regression
SVM: Support vector machine
XGB: Extreme gradient boosting
MLP: Multilayer perceptron
ROC: Receiver operating characteristic
AUC: Area under the ROC curve
Aβ: β Amyloid
NFTs: Neurofibrillary tangles
MRI: Magnetic resonance imaging
ML: Machine learning
MMSE: Mini-Mental State Examination
AI: Artificial intelligence
CDR-SB: Clinical dementia rating-sum of box
ROI: Region of interest
GM: Gray matter
ANOVA: Analysis of variance

## References

[1] Wilson H, Pagano G, Politis M (2019). Dementia spectrum disorders: lessons learnt from decades with PET research. J Neural Transm.

[2] DeTure MA, Dickson DW (2019). The neuropathological diagnosis of Alzheimer’s disease. Mol Neurodegener.

[3] Schöll M, Lockhart SN, Schonhaut DR, O’Neil JP, Janabi M, Ossenkoppele R (2016). PET imaging of tau deposition in the aging human brain. Neuron.

[4] Braak H, Braak E (1991). Neuropathological stageing of Alzheimer-related changes. Acta Neuropathol.

[5] Jack CR, Bennett DA, Blennow K, Carrillo MC, Dunn B, Haeberlein SB (2018). NIA-AA research framework: toward a biological definition of Alzheimer’s disease. Alzheimers Dement.

[6] Rocchi L, Niccolini F, Politis M (2015). Recent imaging advances in neurology. J Neurol.

[7] Politis M (2014). Neuroimaging in Parkinson disease: from research setting to clinical practice. Nat Rev Neurol.

[8] Politis M, Piccini P (2012). Positron emission tomography imaging in neurological disorders. J Neurol.

[9] Márquez F, Yassa MA (2019). Neuroimaging biomarkers for Alzheimer’s disease. Mol Neurodegener.

[10] Kuznetsov IA, Kuznetsov AV (2018). Simulating the effect of formation of amyloid plaques on aggregation of Tau protein. Proc Math Phys Eng Sci.

[11] Braak H, Braak E (1997). Frequency of stages of Alzheimer-related lesions in different age categories. Neurobiol Aging.

[12] Duyckaerts C, Brion JP, Hauw JJ, Flament-Durand J (1987). Quantitative assessment of the density of neurofibrillary tangles and senile plaques in senile dementia of the Alzheimer type. Comparison of immunocytochemistry with a specific antibody and Bodian’s protargol method. Acta Neuropathol.

[13] Baker SL, Maass A, Jagust WJ (2017). Considerations and code for partial volume correcting [18F]-AV-1451 tau PET data. Data Brief.

[14] Cho H, Choi JY, Hwang MS, Kim YJ, Lee HM, Lee HS (2016). In vivo cortical spreading pattern of tau and amyloid in the Alzheimer disease spectrum. Ann Neurol.

[15] Wang L, Benzinger TL, Su Y, Christensen J, Friedrichsen K, Aldea P (2016). Evaluation of tau imaging in staging Alzheimer disease and revealing interactions Between β-amyloid and tauopathy. JAMA Neurol.

[16] Villemagne VL, Fodero-Tavoletti MT, Masters CL, Rowe CC (2015). Tau imaging: early progress and future directions. Lancet Neurol.

[17] Schöll M, Schonhaut D, Lockhart S, Vogel JW, Baker S, Schwimmer H (2015). In vivo braak staging using 18F-AV1451 Tau PET imaging. Alzheimers Dement.

[18] Jack CR, Wiste HJ, Weigand SD, Therneau TM, Lowe VJ, Knopman DS (2017). Defining imaging biomarker cut points for brain aging and Alzheimer’s disease. Alzheimers Dement.

[19] Pellegrini E, Ballerini L, del Hernandez MCV (2018). Machine learning of neuroimaging for assisted diagnosis of cognitive impairment and dementia: a systematic review. Alzheimers Dement Diagn Assess Dis Monit..

[20] Velazquez M, Lee Y, Alzheimer’s Disease Neuroimaging Initiative (2021). Random forest model for feature-based Alzheimer’s disease conversion prediction from early mild cognitive impairment subjects. PLoS ONE.

[21] Li X, Zhou Y, Dvornek NC, Gu Y, Ventola P, Duncan JS (2020). Efficient Shapley explanation for features importance estimation Under uncertainty. Med Image Comput Comput Assist Interv.

[22] Nohara Y, Matsumoto K, Soejima H, Nakashima N (2022). Explanation of machine learning models using Shapley additive explanation and application for real data in hospital. Comput Methods Programs Biomed.

[23] Veitch DP, Weiner MW, Aisen PS, Beckett LA, DeCarli C, Green RC (2022). Using the Alzheimer’s disease neuroimaging initiative to improve early detection, diagnosis, and treatment of Alzheimer’s disease. Alzheimers Dement.

[24] Hu W, Wu F, Zhang Y, Gong CX, Iqbal K, Liu F (2017). Expression of tau pathology-related proteins in different brain regions: a molecular basis of tau pathogenesis. Front Aging Neurosci.

[25] Young CB, Landau SM, Harrison TM, Poston KL, Mormino EC, ADNI (2021). Influence of common reference regions on regional tau patterns in cross-sectional and longitudinal [18F]-AV-1451 PET data. Neuroimage.

[26] Doré V, Krishnadas N, Bourgeat P, Huang K, Li S, Burnham S (2021). Relationship between amyloid and tau levels and its impact on tau spreading. Eur J Nucl Med Mol Imaging.

[27] Goenka N, Tiwari S (2022). Multi-class classification of Alzheimer’s disease through distinct neuroimaging computational approaches using Florbetapir PET scans. Evol Syst.

[28] Shirbandi K, Khalafi M, Mirza-Aghazadeh-Attari M, Tahmasbi M, Kiani Shahvandi H, Javanmardi P (2021). Accuracy of deep learning model-assisted amyloid positron emission tomography scan in predicting Alzheimer’s disease: a systematic review and meta-analysis. Inform Med Unlocked.

[29] Maass A, Landau S, Baker SL, Horng A, Lockhart SN, La Joie R (2017). Comparison of multiple tau-PET measures as biomarkers in aging and Alzheimer’s disease. Neuroimage.

[30] Baker SL, Lockhart SN, Price JC, He M, Huesman RH, Schonhaut D (2017). Reference tissue-based kinetic evaluation of 18F-AV-1451 for tau imaging. J Nucl Med.

[31] Cho SH, Choe YS, Park S, Kim YJ, Kim HJ, Jang H (2020). Appropriate reference region selection of 18F-florbetaben and 18F-flutemetamol beta-amyloid PET expressed in Centiloid. Sci Rep.

[32] Chen J, Li Y, Pirraglia E, Okamura N, Rusinek H, de Leon MJ, Alzheimer’s Disease Neuroimaging Initiative (2018). Quantitative evaluation of tau PET tracers 18F-THK5351 and 18F-AV-1451 in Alzheimer’s disease with standardized uptake value peak-alignment (SUVP) normalization. Eur J Nucl Med Mol Imaging.

[33] Leuzy A, Pascoal TA, Strandberg O, Insel P, Smith R, Mattsson-Carlgren N (2021). A multicenter comparison of [18F]flortaucipir, [18F]RO948, and [18F]MK6240 tau PET tracers to detect a common target ROI for differential diagnosis. Eur J Nucl Med Mol Imaging.

[34] Sabri O, Sabbagh MN, Seibyl J, Barthel H, Akatsu H, Ouchi Y (2015). Florbetaben PET imaging to detect amyloid beta plaques in Alzheimer’s disease: phase 3 study. Alzheimers Dement.

[35] Kotari V, Navitsky M, Southekal S, Kennedy I, Harris T, Lu M (2019). Early tau detection and implications for disease progression. Alzheimers Dement.

[36] Joshi AD (2021). Performance characteristics of amyloid PET with florbetapir F 18 in patients with alzheimer’s disease and cognitively normal subjects. J Nucl Med.

[37] Cho SH (2020). Concordance in detecting amyloid positivity between 18F-florbetaben and 18F-flutemetamol amyloid PET using quantitative and qualitative assessments. Sci Rep.

[38] Feng J, Xu H, Mannor S, Yan S (2014). Robust logistic regression and classification. Adv Neural Inf Proc Sys.

[39] Cortes C, Vapnik V (1995). Support-vector networks. Mach Learn.

[40] Chen T, Guestrin C. XGBoost: A scalable tree boosting system. Proceedings of the 22nd ACM SIGKDD International Conference on Knowledge Discovery and Data Mining. 2016; pp. 785–94.

[41] Hastie T (2022). The elements of statistical learning.

[42] Banerjee K, C. VP, Gupta RR, Vyas K, H A, Mishra B. Exploring Alternatives to Softmax Function. arXiv, 2020. 10.48550/arXiv.2011.11538.

[43] Agarap AF. Deep learning using Rectified Linear Units (ReLU). arXiv, 2020. 10.48550/arXiv.1803.08375.

[44] Gulcehre C, Denil M, Malinowski M, et al. Hyperbolic attention networks. arXiv 2018. 10.48550/arXiv.1805.09786.

[45] Lydia AA, Francis FS. An optimizer for stochastic gradient descent. IJICS 2019;6;566-568.

[46] Duda J. SGD momentum optimizer with step estimation by online parabola model. arXiv 2019. 10.48550/arXiv.1907.07063.

[47] Bloch L, Friedrich CM (2021). Data analysis with Shapley values for automatic subject selection in Alzheimer’s disease data sets using interpretable machine learning. Alzheimers Res Ther..

[48] Lundberg SM, Lee SI. A unified approach to interpreting model predictions. arXiv. 2017. 10.48550/arXiv.1705.07874.

[49] Akiba, Takuya, et al. Optuna: A next-generation hyperparameter optimization framework. Proceedings of the 25th ACM SIGKDD International Conference on Knowledge Discovery & Data Mining 2019; pp. 2623–31.

