# Supplementary figures and images for: Machine learning application for classification of Alzheimer's disease stages using 18F-flortaucipir positron emission tomography
Source: Biomed Eng Online. 2023 Apr 29;22:40. doi: 10.1186/s12938-023-01107-w (PMC10149022; doi:10.1186/s12938-023-01107-w)

## Slide 1
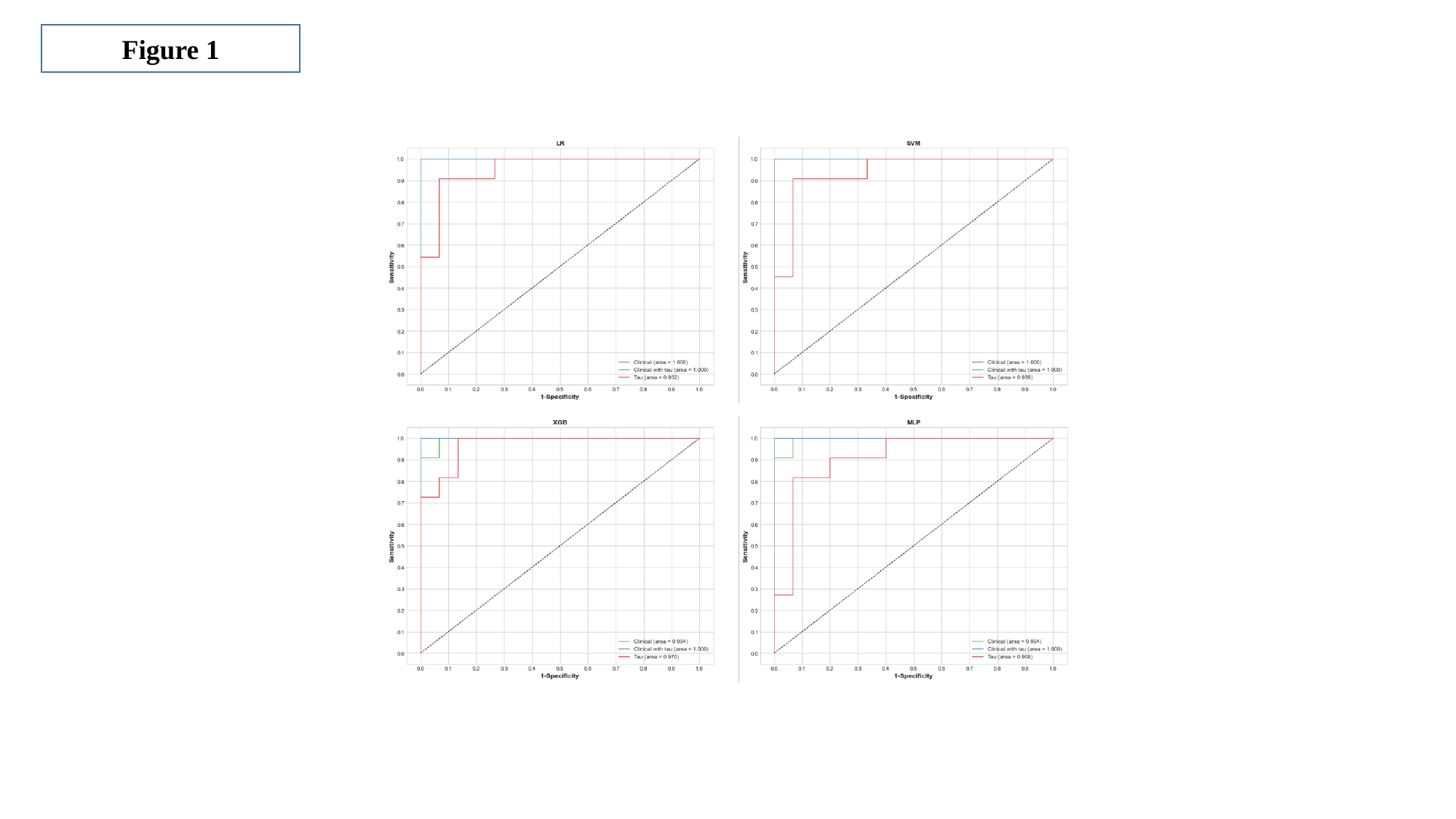

Figure 1

## Slide 2
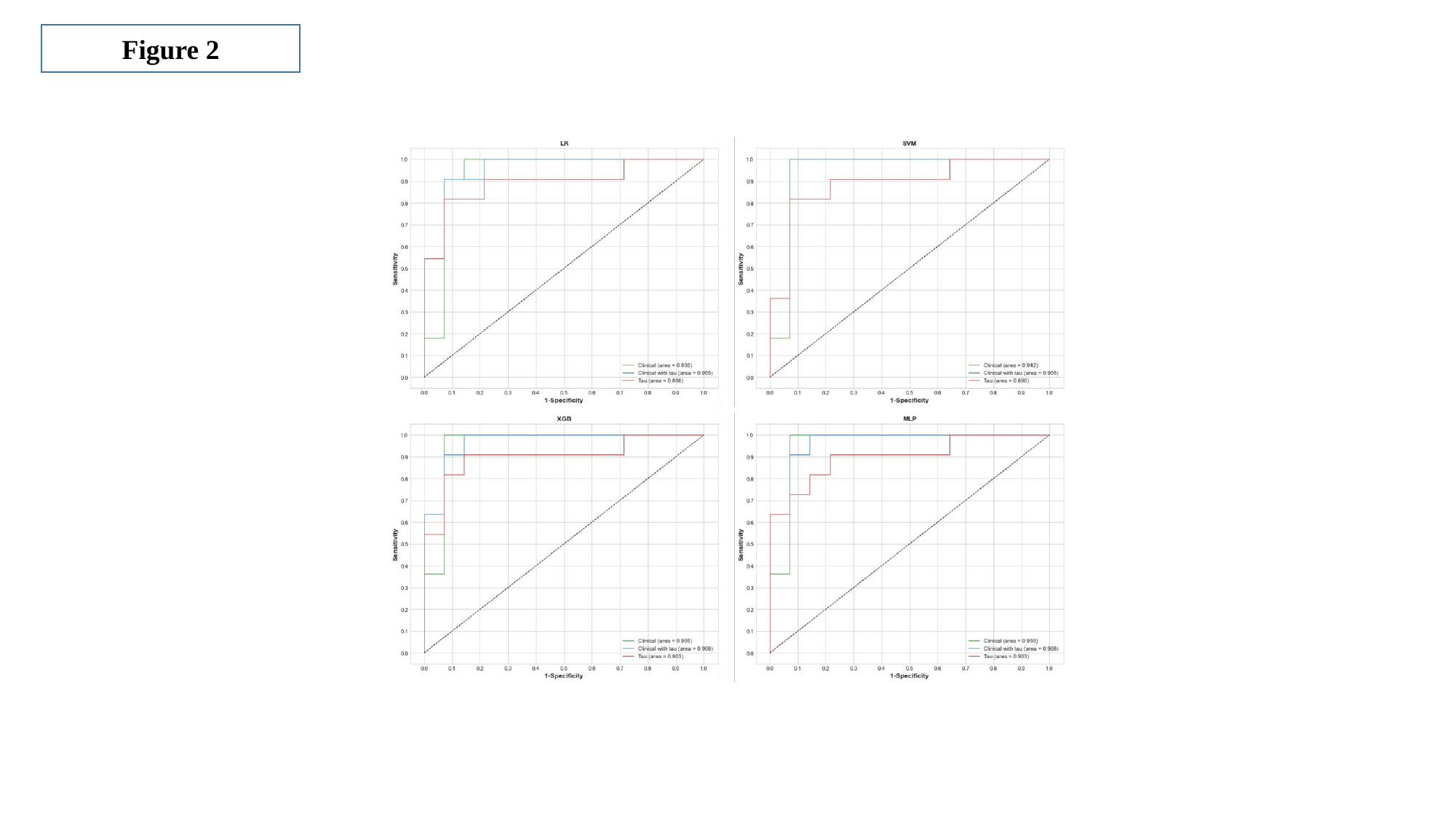

Figure 2

## Slide 3
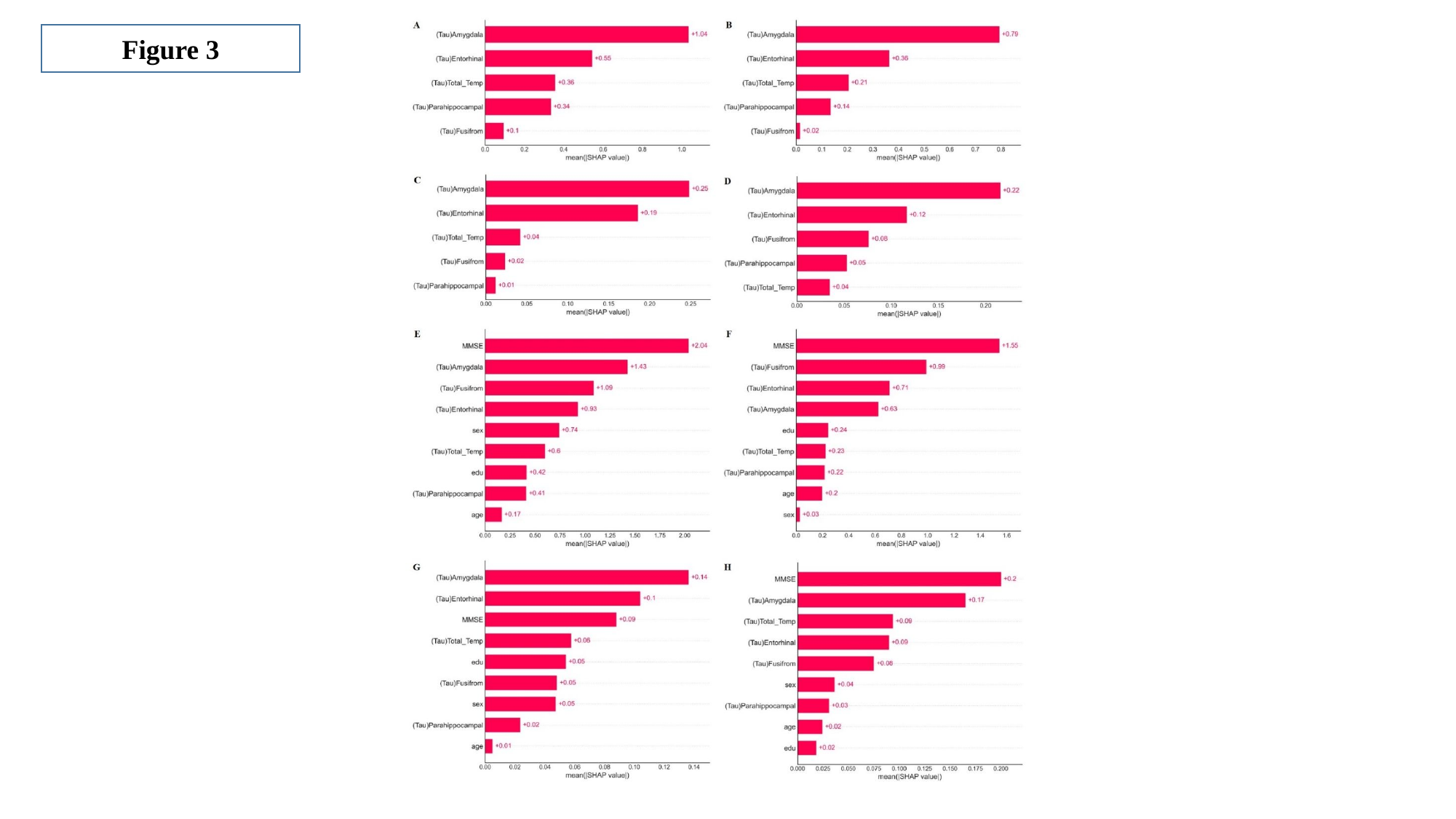

Figure 3

## Slide 4
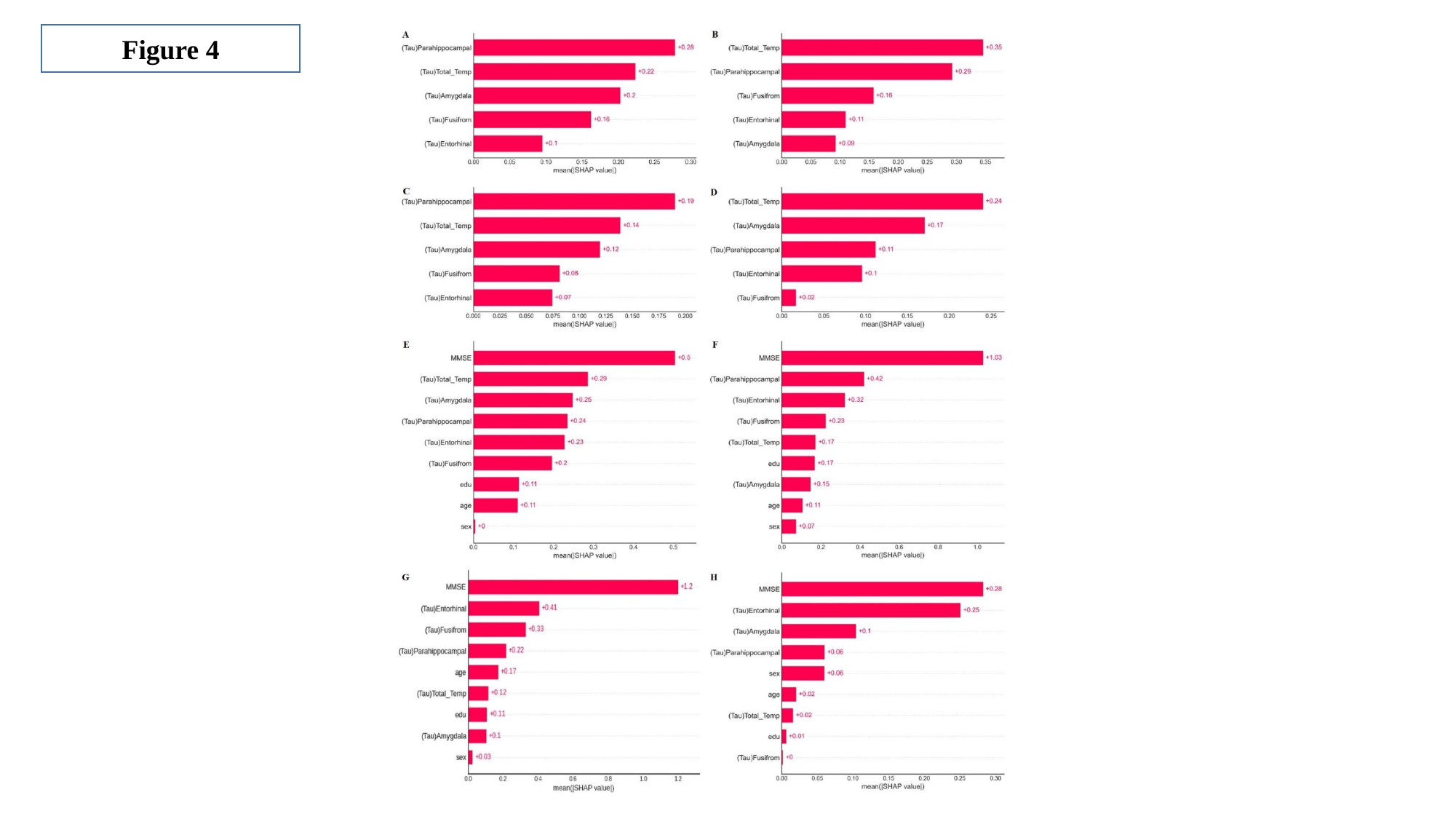

Figure 4

Supplement: Supplementary file 1 — Additional file 1. Fig. 1: Receiver operating characteristic curve for classification between cognitively unimpairment and Alzheimer’s dementia. Fig. 2: Receiver operating characteristic curve for classification between mild cognitive impairment and Alzheimer’s dementia. Fig. 3. In between cognitive unimpairment and Alzheimer's disease, feature importance results based on the explainable Shapley Additive Explanations method. A–D Are the results for the importance of the tau standard uptake value ratio (SUVR) features, considering the trade-offs between the features. e–h are the results for clinical variables with tau SUVR features. Fig. 4. In between mild cognitive impairment and Alzheimer's disease, feature importance results based on the explainable Shapley Additive Explanations method. A–D Are the results for the importance of the tau standard uptake value ratio (SUVR) features, considering the trade-offs between the features. e–h are the results for clinical variables with tau SUVR features. [file 12938_2023_1107_MOESM1_ESM.pptx]
